# Reproductive Health Challenges Among Adolescents in Eastern Europe: Evidence from a Romanian Tertiary Hospital

**DOI:** 10.3390/healthcare14040550

**Published:** 2026-02-23

**Authors:** Mihaela-Camelia Tîrnovanu, Elena Țarcă, Elena Cojocaru, Vlad-Gabriel Tîrnovanu, Ștefan-Dragoș Tîrnovanu, Awad Dmour, Monica Holicov, Corina-Cristina Zamfir, Sorina-Cristiana Gheorghiu, Roxana Ana Covali, Gabriel Costăchescu, Viorel Țarcă

**Affiliations:** 1 Grigore T. Popa University of Medicine and Pharmacy Iasi, 700115 Iași, Romania; mihaela.tirnovanu@umfiasi.ro (M.-C.T.); tarca.elena@umfiasi.ro (E.Ț.); elena2.cojocaru@umfiasi.ro (E.C.); dmour-awad@umfiasi.ro (A.D.); monica.holicov@umfiasi.ro (M.H.); ana.covali@umfiasi.ro (R.A.C.); gabriel.costachescu@umfiasi.ro (G.C.); 2 “Cuza Voda” Obstetrics-Gynecology Clinic Hospital, 700038 Iasi, Romania; corina.zamfir235@icloud.com (C.-C.Z.); sorina.gheorghiu98@yahoo.ro (S.-C.G.); 3 St. Josef Hospital, 65189 Wiesbaden, Germany; vlad.tirno@gmail.com; 4 Faculty of Medicine, Apollonia University, Păcurari Street nr. 11, 700511 Iasi, Romania; viorel.tarca@univapollonia.ro

**Keywords:** adolescent pregnancy, prenatal care, maternal education, neonatal outcomes, reproductive health disparities, early childbearing, pelvic development

## Abstract

**Background**: Adolescent pregnancy remains a major global public health issue, often linked to socioeconomic and educational disparities rather than biological immaturity. This study aimed to identify sociodemographic factors associated with adolescent pregnancies and to evaluate their impact on maternal and neonatal outcomes in a tertiary hospital in Northeastern Romania. **Methods**: A retrospective analysis was conducted at the “Cuza Vodă” Obstetrics-Gynecology Clinic Hospital, Iași, over two periods: 2013–2017 and January–October 2025. Records of 637 mothers aged <20 years were reviewed. Variables included age, education, prenatal monitoring, gestational age, delivery mode, neonatal outcomes, and obstetric complications. Statistical analyses were performed using SPSS v26, employing ANOVA, Welch ANOVA, and post hoc tests (*p* < 0.05). Ethical approval was obtained from the institutional ethics committee. **Results**: The mean maternal age was 17.26 ± 1.5 years, with 82.6% from rural areas. Most had only primary or lower secondary education. Fully monitored pregnancies were associated with significantly higher birth weights (mean = 3249 g) compared with unmonitored pregnancies (mean = 3009 g; *p* < 0.001). Infants of mothers with low education had the lowest mean birth weights (2963 g; *p* = 0.002). Preterm births represented 14.3% of cases, and cesarean deliveries accounted for 34.5%. A slight but significant increase in maternal age was observed between 2013–2017 and 2025 (*p* < 0.001), suggesting delayed adolescent childbearing. **Conclusions**: Low educational attainment and inadequate prenatal monitoring remain major determinants of adverse neonatal outcomes among adolescent mothers. Comprehensive sexual education, improved prenatal care accessibility, and social support programs are essential to reduce adolescent pregnancy rates and improve reproductive health in Romania.

## 1. Introduction

Adolescent reproductive health remains a critical public health concern in Eastern Europe, where persistent socioeconomic disparities, limited access to preventive services, and inconsistent health education continue to shape vulnerable reproductive trajectories, as reflected by clinical experience in Romanian tertiary care settings. The United Nations created the Sustainable Development Goals (2015–2030), which include the overarching objective of “good health and well-being” for everyone. The list consists of 17 items, including gender equality (item 5), reduced inequalities (item 10), good health and well-being (item 3), and high-quality education (item 4) [1]. These four stated objectives highlight the need for focused work in this area and are closely related to the unresolved problem of teenage pregnancy. Adolescents account for about 11% of all births worldwide, which is a noteworthy percentage. The majority of the 21 million adolescent pregnancies that occur worldwide each year end in birth, according to data from the World Health Organization (WHO). In addition, pregnancy rates among adolescents have decreased from 64.5% per 1000 women in 2000 to 42.5% in 2021 [2].

Pregnancy among adolescents (ages 10 to 19) poses several difficulties, including health concerns, social and economic problems, such as school dropout, and limited long-term career options that can result in long-term financial hardship. The absence of easily accessible, nonjudgmental healthcare treatments is another concern pertaining to teenage reproductive health, with stigma and anxiety about confidentiality violations serving as significant obstacles. Many adolescent mothers rely on public assistance programs because they live in poverty. Early pregnancy can result in social stigmatization, a decline in social standing, and even violence or rejection from friends and family.

Age-appropriate sexual and reproductive health information and services are not readily available to many teenagers. Significant obstacles to getting care include stigma, fear of confidentiality violations, and unwelcoming or judgmental health providers. Adolescents may be compelled to look for risky alternatives if they encounter negative attitudes from healthcare professionals. Because of this, many of them either do not have access to prenatal care at all or do not visit the doctor for proper prenatal care. Abortion, which is frequently dangerous in low- and middle-income nations, ends a large percentage of unwanted adolescent pregnancies worldwide.

Teenagers are more likely to experience early labor, extended labor, and miscarriages [3]. Pregnancy problems, including pre-eclampsia, pregnancy-related hemorrhage, anemia, difficult labor, premature rupture of membranes, low birth weight, premature birth, stillbirth, and congenital defects, are common among teenagers who are pregnant [4,5]. They may not have fully formed bodies, which makes them more susceptible to problems. Because they are less likely to be physically mature enough to maintain a successful pregnancy or deliver a baby, girls under the age of 15 are especially vulnerable. Rapid recurrent pregnancy is a worry for young moms in certain contexts, posing additional dangers to the mother and child. Risks for girls between the ages of 15 and 19 are more closely linked to socioeconomic variables than to the biological impacts of aging. Adolescent pregnancy remains one of the main causes of intergenerational cycles of poverty and poor health [6].

Within the SDG framework, adolescent pregnancy is a cross-cutting indicator of inequities that simultaneously affect health (SDG 3), education (SDG 4), and gender equality (SDG 5). SDG 3.7 highlights the need for universal access to sexual and reproductive health services and information, SDG 5.6 emphasizes sexual and reproductive health and rights, and SDG 4 focuses on educational participation and attainment—each of which is impacted by early pregnancy and, in turn, influences its risks. Although our retrospective clinical dataset cannot quantify SDG indicators at the population level, it can document vulnerable profiles encountered in routine care (e.g., limited schooling, younger age groups, and repeat adolescent births), thereby informing local prevention approaches and service organization consistent with the SDG agenda [1,2].

The rationale for examining adolescent pregnancy in Romania extends beyond counting births: it requires understanding who the affected adolescents are in clinical practice and what modifiable vulnerabilities cluster in this group. In settings where routinely collected medical records do not capture household income, parental education, or broader social determinants, education level and residence may still function as pragmatic proxies of vulnerability that are actionable in service planning. Describing such profiles in a high-volume maternity center can help bridge the gap between broad policy goals and the realities encountered at the point of care. Programs should be put in place, in our opinion, to lower the number of adolescent pregnancies, assist them in their socioeconomic endeavors, and help them integrate into society. This study aimed to describe the socio-demographic and obstetric profile of adolescent mothers who delivered in a tertiary maternity hospital in Romania, with emphasis on education level, residence, age distribution, and repeat adolescent births. We further aimed to compare patterns across the study periods available in our records and to discuss implications for prevention strategies and postpartum counseling within the constraints of routinely collected clinical data.

## 2. Materials and Methods

A retrospective study was conducted in two periods, between January 2013 and December 2017 and between January and October 2025. It was conducted in “Cuza Vodă” Obstetrics-Gynecology Clinic Hospital from Iași, a tertiary hospital in the northeast part of Romania. We intended to compare two distinct periods to illustrate changes in case volume and characteristics at the institutional level. We analyzed the records of all patients who delivered in our hospital, with an age of less than 20 years. Data were extracted retrospectively from routinely collected obstetric and neonatal medical records. The variables included in the analysis were those consistently documented in standard hospital charts (e.g., age, background, completed studies, degree of pregnancy follow-up by a doctor, gestational age at birth, mode of delivery, newborn weight, Apgar score at birth, possible pregnancy complications, and administration of oxytocin during labor and peripartum antibiotics). Pregnancies that completed 37 and above weeks of gestation were considered term pregnancies, while pregnancies of less than 37 weeks were considered preterm pregnancies. A pregnancy that completed 24 weeks or more, according to the last menstrual period, or a newborn with a birth weight of over 500 g, was considered a delivery and included in the study.

Variables capturing household income, parental education/occupation, and detailed social vulnerability measures were not systematically available in medical records and were therefore not included to avoid extensive missingness and potential misclassification. Consequently, our analysis focuses on clinically documented proxies and emphasizes descriptive, setting-specific interpretation.

To conduct the study, ethical approval was obtained from the Scientific Research Ethics Committee of the hospital.

Data from our study were analyzed using IBM SPSS Statistics for Windows, Version 26.0 (IBM Corp., Armonk, NY, USA). Descriptive statistics (means, standard deviations, frequencies, and percentages) were computed to summarize the sample characteristics.

Consistent with the descriptive aim of this retrospective study, we primarily applied univariate statistical analyses to summarize and compare sociodemographic, obstetric, and neonatal characteristics between the study periods. Therefore, following the descriptive analysis of the study variables, an inferential statistical approach was employed to examine whether the observed differences between groups were statistically meaningful. To assess variations in continuous outcomes across categories of maternal educational level, a one-way Analysis of Variance (ANOVA) was conducted.

Before applying ANOVA, its underlying assumptions were examined. These include the independence of observations, approximate normal distribution of the continuous variables within each group, and homogeneity of variances across groups. Given the relatively large sample sizes in all educational subgroups, the Central Limit Theorem supports the robustness of ANOVA to moderate deviations from normality. Homogeneity of variances was assessed using Levene’s test. When this assumption was met, the standard one-way ANOVA was applied; when violated, the Welch ANOVA—an adjusted, more robust variant—was used. In cases where the overall ANOVA indicated statistically significant differences, post hoc pairwise comparisons (Bonferroni or Games–Howell, depending on variance equality) were performed to identify which specific groups differed from each other. The results are considered statistically significant at the *p* < 0.05 level.

## 3. Results

A total of 637 adolescent mothers (<20 years old) and their newborns were included in the analysis. The mothers’ mean age at delivery was 17.26 years (SD = 1.49, range = 12–20). Most participants lived in rural areas (approximately 82.57%) and had completed primary or lower secondary education.

To provide an overview of the sample characteristics, descriptive statistics were calculated for all maternal and neonatal variables included in the study. The results, summarized in Table 1, highlight the general profile of adolescent mothers and their newborns.

**Figure 1:**
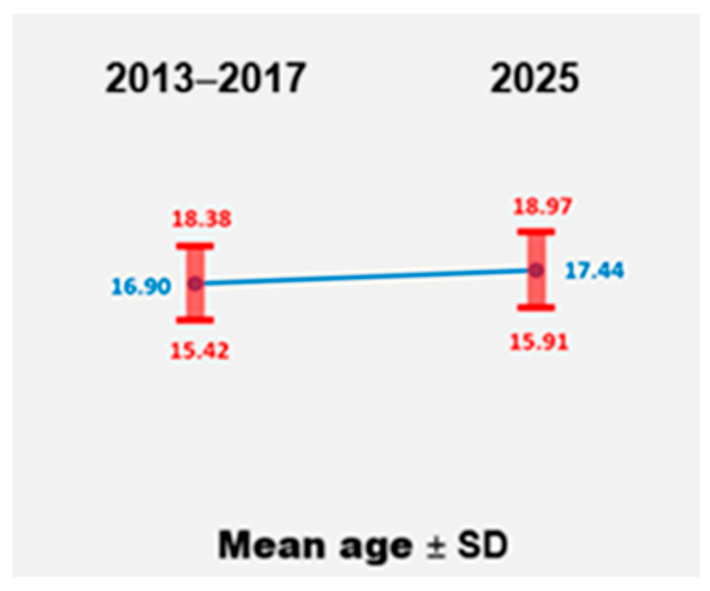
Group means and standard deviations for maternal age in adolescent mothers in 2025 vs. 2013–2017 (at first birth).

Age at first birth is a critical indicator of reproductive health and socio-economic development, as it is closely associated with maternal and child health outcomes. Comparing the mean age of adolescent mothers in 2025 with the cumulative mean from 2013 to 2017 is essential for understanding temporal changes in adolescent reproductive behavior. Such analyses help identify subtle demographic shifts that may reflect broader social, educational, economic, or healthcare-related trends. Even small increases in the age at first birth within the adolescent group can suggest improvements in access to contraception, reductions in early unintended pregnancies, or delayed sexual debut. The mean age of adolescent mothers (<20 years) in 2025 was compared with the cumulative mean from 2013 to 2017 using an independent samples *t*-test (Figure 1).

Descriptive statistics indicated that adolescent mothers in 2025 were slightly older (M = 17.44, SD = 1.53, n = 157) compared with those from the previous group in 2013–2017 (M = 16.90, SD = 1.48, n = 347). Levene’s test confirmed homogeneity of variances (F = 0.825, *p* = 0.364), so the assumption of equal variances was met.

The independent samples *t*-test showed a statistically significant difference between the two periods, t(635) = 3.773, *p* < 0.001, with a mean difference of 0.54 years (SE = 0.144). The 95% confidence interval for the difference ranged from 0.260 to 0.826 years, indicating that the increase is both statistically significant and unlikely to be due to random variation.

These findings indicate that adolescent mothers in 2025 were on average slightly older than those in the preceding decade. Although the difference (approximately six months) may appear modest, it reflects a statistically reliable upward shift and may suggest improving trends in adolescent reproductive health. This pattern may point to delayed childbearing within the adolescent population, potentially associated with better access to education, contraception, and public health interventions aimed at reducing early teenage pregnancies.

Geographic disparities, particularly between urban and rural populations, can reflect differences in access to education, healthcare services, and family planning resources. We conducted a comparison between the age at first birth among women from urban and rural areas to assess potential differences in reproductive timing across geographic settings.

**Figure 2:**
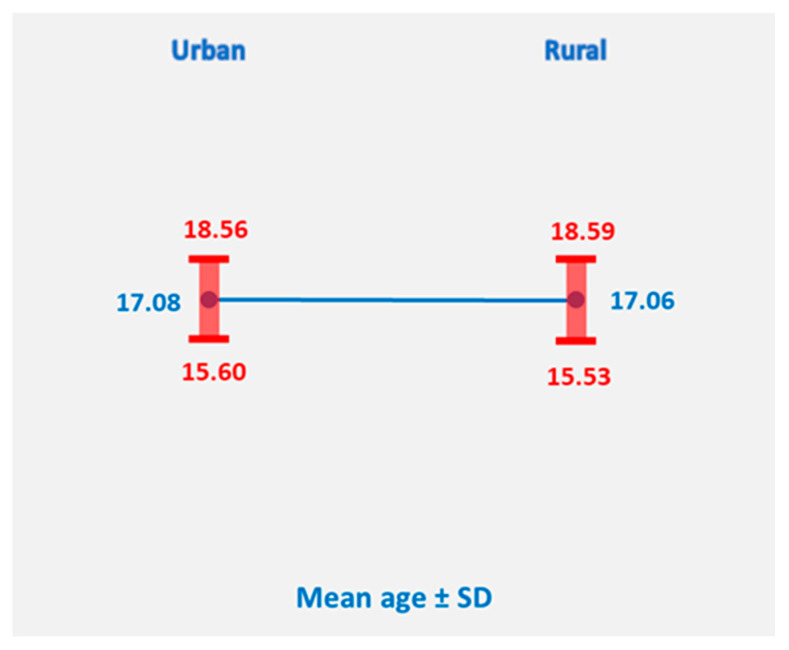
Distribution of mean age at first birth in urban and rural cohorts.

Descriptive statistics indicated that women from urban areas (n = 89) had a mean age at first birth of 17.08 years (SD = 1.36), while women from rural areas (n = 415) had a mean age of 17.06 years (SD = 1.55). The difference between the two groups was minimal, suggesting that, in this cohort, geographic environment had little influence on the timing of first childbirth (Figure 2). Although national reports often emphasize rural disadvantage, our cohort showed a near-balanced distribution, suggesting that in our catchment area, adolescent pregnancy is not exclusively a rural phenomenon, and interventions should address both settings. These findings may reflect similar socio-cultural and reproductive patterns across urban and rural settings in the study population.

An independent-samples *t*-test was conducted to compare the age at first birth for women from urban and rural areas. Levene’s test indicated that the assumption of equal variances was met (F = 2.548, *p* = 0.111). The *t*-test showed no statistically significant difference between the two groups, t(502) = 0.090, *p* = 0.928, with a mean difference of 0.016 years (95% CI: −0.332 to 0.364).

These results suggest that, in this cohort, geographic environment (urban vs. rural) did not meaningfully influence the age at first birth, indicating broadly similar reproductive timing across settings.

Most pregnancies were singleton and spontaneous, with a mean gestational age at admission of 38.03 weeks (SD = 2.21). The mean birth weight was 3077.03 g (SD = 560.80 g, range = 670–4550 g). Approximately 10.99% of infants were classified as low birth weight (<2500 g), and 14.29% were identified as preterm deliveries (<37 weeks).

Regarding complications during pregnancy, intrauterine growth restriction (IUGR) was documented in 9.58% of cases, amnionitis in 5.49%, and hypertension during pregnancy in 3.30%. Cesarean section accounted for 34.54% of all deliveries, while the remaining 65.46% were vaginal births. From the total number of 220 adolescents with birth by cesarean section, the indication for this way of delivery was cephalopelvic disproportion for 28 (12.72%) of them, and a narrow maternal pelvis for 15 (6.81%). In our study, we had only one case of an 18-year-old woman with right sacroiliac joint dysfunction, which manifested in the 7th day postpartum, with partial functional impotence of the lower limb, which resolved within 4 days after anti-inflammatory treatment. She delivered, by cesarean section, a newborn of 4100g after a trial of labor.

The mean Apgar score at 1 min was 8.15 (SD = 1.61), indicating generally favorable neonatal outcomes.

The overall mean age of the mothers in the sample (N = 637) was 17.26 years (SD = 1.50). Age differed visibly across educational groups. Mothers with the lowest educational attainment (“At most primary school,” n = 204) had a mean age of 16.78 years (SD = 1.70), whereas those with a middle school education (n = 347) had a slightly higher mean age of 17.24 years (SD = 1.34). The oldest group consisted of mothers with high school or vocational training (n = 86), who had a mean age of 18.45 years (SD = 0.76). It is important to mention that 30 teenage mothers from our study did not go to school at all, and 11 of them delivered in 2025. It is hard to believe that in the era of routine use of computers and artificial intelligence, we have teenage girls giving birth and no education whatsoever.

In the study group, we had adolescents on their second, third, or even fourth births. Examining the gravidity–parity distribution among women with limited formal education provides critical insight into reproductive behavior patterns that are closely linked to maternal and neonatal health outcomes.

Table 2 illustrates the distribution of gravidity (number of pregnancies) and parity (number of live births) among a clinical cohort of 204 women who completed at most primary school during the period of our study. Most women were experiencing their first pregnancy and had no previous live births (parity 1), accounting for 144 of the 204 participants. Higher gravidity and parity combinations were progressively less frequent, with only a small number of women having three or more pregnancies or live births. These data highlight the reproductive patterns within this educational subgroup and can provide insight into maternal health needs, family planning utilization, and potential risks associated with higher parity in women with limited formal education.

Birth weight also displayed variation across educational levels. The mean birth weight for the entire sample was 3077.03 g (SD = 560.80). Infants born to mothers with “At most primary school” education (n = 204) had the lowest mean birth weight (2963.14 g, SD = 565.35). The middle-school group (n = 347) showed a higher mean birth weight of 3122.51 g (SD = 548.25), while the highest mean was observed among infants of mothers with high school or vocational education (n = 86), at 3163.72 g (SD = 564.94).

**Figure 3:**
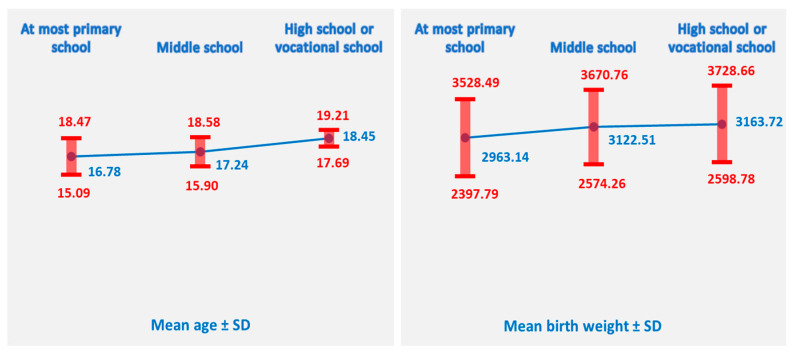
Maternal age and neonatal birth weight across educational levels.

Overall, both maternal age and neonatal birth weights appeared to increase with higher levels of maternal educational attainment (Figure 3).

These patterns provided the rationale for conducting inferential analyses to determine whether the observed differences between groups were statistically significant. Consequently, a one-way Analysis of Variance (ANOVA) was performed to evaluate group differences in maternal age and birth weight across the three educational levels.

Before conducting the ANOVA, the assumption of homogeneity of variances was assessed using Levene’s test. For maternal age, Levene’s test indicated statistically significant differences in variances across the three educational groups (Based on Mean: F(2, 634) = 30.556, *p* < 0.001). The significant result suggests that the assumption of equal variances was violated for this variable. Consequently, the standard one-way ANOVA is not appropriate for maternal age, and the more robust Welch ANOVA was applied for this comparison. In contrast, Levene’s test for neonatal birth weight yielded a non-significant result (Based on Mean: F (2, 634) = 0.055, *p* = 0.947), indicating that the variances did not differ significantly across educational levels. Therefore, the assumption of homogeneity of variances was met for birth weight, and the standard one-way ANOVA was used for this analysis.

The ANOVA revealed a statistically significant effect of maternal educational level on neonatal birth weight, F (2, 634) = 6.485, *p* = 0.002. This finding indicates that the mean birth weight differed across the three educational categories.

Inspection of group means showed a clear pattern in which birth weight increased with higher maternal educational attainment: infants of mothers with “At most primary school” education had the lowest mean birth weight (2963 g), while those born to mothers with “Middle school” and “High school or vocational school” education had progressively higher mean values (3123 g and 3164 g, respectively). These results suggest that higher maternal education may be associated with more favorable birth outcomes.

A Bonferroni post hoc test was conducted to examine pairwise differences in mean birth weight among mothers with different education levels. Infants born to mothers with at most primary school education had significantly lower birth weights compared to those born to mothers with middle school education (Mean Difference = −159.37 g, *p* = 0.004) and high school or vocational school education (Mean Difference = −200.58 g, *p* = 0.016). There was no significant difference in birth weight between infants of mothers with middle school and high school or vocational school education (Mean Difference = −41.21 g, *p* = 1.000).

These findings indicate that lower maternal education is associated with lower infant birth weight. Specifically, infants of mothers with only primary education are at higher risk of lower birth weight compared to those whose mothers attained at least middle or high school education. The difference between middle- and high-school-educated mothers was not statistically significant, suggesting that the effect of education on birth weight is most pronounced at the lowest education level.

The Welch test showed a highly significant effect of maternal educational level on maternal age, Welch’s F (2, 295.333) = 90.317, *p* < 0.001. This result confirms mean maternal age differs substantially across the three educational categories.

Consistent with the descriptive findings, mothers with “High school or vocational school” education were significantly older than those in the “Middle school” and “At most primary school” groups, while the youngest mothers were those with the lowest educational attainment. These results indicate a strong positive association between maternal education and maternal age among adolescent mothers.

Because the assumption of homogeneity of variances was violated for maternal age, pairwise group comparisons were conducted using the Games–Howell post hoc test, which does not assume equal variances or equal sample sizes. The results demonstrated that all three educational groups differed significantly from one another (*p* < 0.05).

Specifically, mothers with “At most primary school” education were significantly younger than those with “Middle school” education (mean difference = −0.46 years, *p* = 0.003) and substantially younger than those with “High school or vocational school” education (mean difference = −1.67 years, *p* < 0.001). Likewise, mothers in the “Middle school” group were significantly younger than those in the “High school or vocational school” group (mean difference = −1.21 years, *p* < 0.001).

These findings confirm a clear and graded pattern: maternal age increases progressively with higher levels of educational attainment. The largest gap—exceeding 1.6 years—was observed between the lowest and highest educational groups, highlighting a substantial developmental and sociodemographic difference across maternal education levels within this adolescent population.

To examine the relationship between maternal medical monitoring and key maternal and neonatal outcomes, descriptive statistics were computed for maternal age and infant birth weight across three monitoring categories: not monitored (n = 422), partially monitored (n = 79), and fully monitored (n = 136). This analysis provides a preliminary overview of potential differences in maternal and infant characteristics associated with the level of prenatal follow-up.

The mean age of adolescent mothers varied slightly across medical monitoring groups: those who were not monitored had a mean age of 17.07 years (SD = 1.49), fully monitored mothers averaged 17.54 years (SD = 1.51), and partially monitored mothers were slightly older at 17.78 years (SD = 1.29).

**Figure 4:**
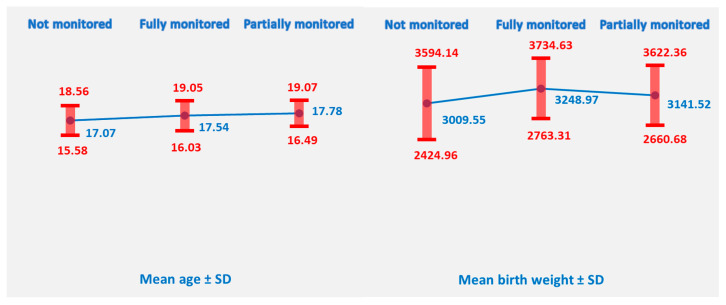
Maternal age and neonatal birth weight across the monitoring categories.

Similarly, infant birth weight differed across the monitoring categories. Infants born to not monitored mothers had the lowest mean birth weight of 3009.55 g (SD = 584.59), those of partially monitored mothers during pregnancy averaged 3141.52 g (SD = 480.84), and the highest mean birth weight was observed in infants of fully monitored mothers during pregnancy at 3248.97 g (SD = 485.66) (Figure 4).

Before conducting the ANOVA, Levene’s test was performed to assess the assumption of homogeneity of variances for maternal age and infant birth weight across the three maternal monitoring groups. For maternal age, Levene’s test was not significant, F (2,634) =1.60, *p* = 0.202, indicating that the assumption of equal variances was met.

For infant birth weight, Levene’s test approached significance but did not reach the threshold, F (2,634) = 2.70, *p* = 0.068, suggesting that the variances across monitoring groups can be considered approximately equal.

The results support the use of standard one-way ANOVA for both maternal age and infant birth weight, as the assumption of homogeneity of variances is satisfied.

A one-way ANOVA was conducted to examine differences in maternal age and infant birth weight across the three maternal monitoring groups (not monitored, partially monitored, and fully monitored). The analysis revealed a significant difference in maternal age among the groups, F (2,634) =11.08, *p* < 0.001. This indicates that mothers’ ages vary depending on their level of medical monitoring. Post hoc comparisons using Bonferroni adjustment indicated that mothers who were not monitored were significantly younger than those who were fully monitored (mean difference = −0.47 years, *p* = 0.004) and partially monitored (Mean Difference = −0.72 years, *p* < 0.001). There was no significant difference in age between fully monitored and partially monitored mothers (Mean Difference = −0.25 years, *p* = 0.702). These results suggest that younger adolescent mothers are less likely to receive full or partial medical monitoring, whereas slightly older adolescents tend to be more fully monitored.

Similarly, there was a significant effect of maternal monitoring on infant birth weight, F (2,634) =10.26, *p* < 0.001. Infants born to mothers in different monitoring categories showed statistically significant differences in mean birth weight. These results suggest that both maternal age and infant birth weight are associated with the level of maternal medical monitoring, justifying further post hoc comparisons to identify which groups differ significantly. Bonferroni post hoc tests showed that infants of fully monitored mothers had significantly higher birth weights than those of not monitored mothers (mean difference = 239.42 g, *p* < 0.001). There was no significant difference between partially monitored and not monitored infants (mean difference = 131.97 g, *p* = 0.156) or between fully monitored and partially monitored infants (mean difference = 107.45 g, *p* = 0.510).

These findings indicate that complete maternal monitoring is associated with higher infant birth weight, while partial monitoring does not differ significantly from either extreme. This underscores the importance of full prenatal follow-up for optimal neonatal outcomes in adolescent mothers.

## 4. Discussion

Our findings should be interpreted in light of the variables available in routine clinical documentation. The associations observed with education level and residence are presented as descriptive patterns within our cohort and should not be construed as direct evidence of underlying causal mechanisms such as household poverty, intergenerational transmission, or minority status—factors that likely matter but were not systematically measurable in our dataset. We therefore frame broader explanations as plausible contextual hypotheses supported by the external literature, while keeping the core conclusions grounded in the observed clinical and socio-demographic profile.

Still, national data indicate divergent trends in adolescent births in Romania. UNICEF reports that births among girls aged 10–14 years increased markedly over the past three decades, while births among adolescents aged 15–19 years decreased overall, albeit with fluctuations. The rise in the 10–14 age group is particularly alarming and remains insufficiently explained, highlighting the need for further research [7]. In our tertiary maternity center, we did not observe a reduction in adolescent deliveries; 211 adolescent deliveries were recorded in 2025, compared with 426 in 2013–2017 (total), suggesting that adolescent pregnancy continues to represent a tangible clinical burden in our setting.

The mean maternal age in our sample was 17.26 years, with deliveries occurring as early as age 12. Although adolescent pregnancy before age 15 is considered rare and high-risk [8], 83 such cases were recorded, indicating a concerning pattern with a noticeable increase in recent years. Although the sample size is limited, this trend warrants further monitoring and analysis. A statistically significant increase in the mean age of adolescent mothers between 2013–2017 and 2025 (from 16.90 to 17.44 years) may suggest delayed childbearing within the adolescent population. While modest, this trend could reflect improving access to reproductive health services, contraception, or education, as observed in other European settings [9].

Level of education emerged as a key factor associated with maternal age, parity, and neonatal outcomes. Mothers with higher education levels were significantly older and more likely to have infants with higher birth weights. These findings align with global evidence indicating that adolescent pregnancy is inversely associated with years of schooling [10] and that lower education levels are linked to earlier childbearing, lower birth weight, and higher parity [11]. Among mothers with only primary education, a subgroup analysis revealed repeated pregnancies by age 19, underscoring the role of education in both preventing early childbearing and delaying subsequent births.

Access to prenatal care remains a critical determinant of maternal and neonatal health. In our study, over two-thirds of adolescent mothers had no or only partial prenatal monitoring. Infants born to fully monitored mothers had significantly higher birth weights compared to those born to mothers with no prenatal care, even after adjusting for maternal age differences. This pattern has been reported in other studies, where lack of autonomy, fear of disclosure, and limited health literacy contribute to poor antenatal attendance among younger adolescents [12]. Also, these results support previous findings that adequate prenatal care is associated with a reduced risk of low birth weight and improved neonatal outcomes [13,14,15].

Educational attainment was inversely associated with the number of pregnancies, with 204 women who had completed only primary school experiencing multiple births. This aligns with prior studies indicating that limited education is a consistent risk factor for adolescent pregnancy and repeated childbearing [16].

The rate of cesarean deliveries in adolescent pregnancies remains a point of clinical interest. In our study, 34.54% of adolescent deliveries were cesarean sections. Although this rate appears elevated, it must be interpreted in the context of our institution’s overall cesarean rate of 65%. The literature reports mixed findings on cesarean section rates among adolescents, with some studies reporting higher rates due to physiological immaturity [17], while others report lower rates [18]. Our data suggest that adolescent pregnancy per se may not be a major determinant of cesarean section, particularly in tertiary care settings with high surgical rates overall.

Adolescent anatomical development, particularly of the pelvis, may influence obstetric outcomes. The timing and pace of pelvic growth are heterogeneous and influenced more by biological maturity than chronological age. Research has shown that the dimensions of the birth canal remain reduced during the initial three years after menarche, only reaching greater maturity by approximately age 18 [19]. Specifically, the internal acetabular and pubic regions continue to develop more significantly during adolescence than other skeletal pelvic regions [20]. This suggests a unique and interdependent relationship between pelvic growth and reproductive maturation. In our study, we had only 28 (12.72%) adolescents with cephalopelvic disproportion caused by large fetuses, and 15 (6.81%) of them had a narrow pelvis, indicating that anatomical immaturity may contribute to delivery complications among younger adolescents. However, these observations must be interpreted with caution due to the retrospective nature of the study and the lack of standardized pelvic assessments across all patients.

Musculoskeletal complications such as pubic symphysis diastasis and sacroiliac joint dysfunction can be observed after vaginal delivery. Sacroiliac joint dysfunction, often associated with factors such as fetal macrosomia, instrumental delivery, multiparity, or rapid labor, typically resolves spontaneously within 3 to 6 months postpartum in over 90% of affected individuals [21,22]. Only one case of postpartum sacroiliac dysfunction was documented in our study.

In terms of neonatal outcomes, 10.99% of infants were classified as low birth weight (<2500 g), and 14.29% of births were preterm. These figures are lower than those reported in some international adolescent cohorts [23,24] but may reflect our hospital’s tertiary-level care. Although some evidence links low birth weight and preterm birth with poor prenatal care and physiological immaturity [25,26], causality cannot be inferred from this retrospective study. Notably, our data show that over two-thirds of adolescent mothers received incomplete or no prenatal care, reinforcing existing evidence on the association between limited antenatal monitoring and adverse outcomes.

The intergenerational nature of adolescent pregnancy was suggested by self-reported maternal age data. Many participants indicated that their own mothers had also given birth during adolescence. Although this observation supports the existing literature on familial transmission of early pregnancy [27,28], further studies with formal genealogical or longitudinal designs are needed.

Limited adherence to postpartum contraceptive counseling was also observed. Despite access to medical counseling, many adolescents did not initiate contraception, leading to repeat pregnancies before the age of 19. This underscores the challenges in translating contraceptive education into behavioral change, particularly in vulnerable adolescent populations. Our observation of limited contraceptive use, including among adolescents with repeat pregnancies, highlights a gap between information provision and effective service uptake. A feasible response may include standardized postpartum counseling protocols, direct linkage to family planning services before discharge, confidential follow-up options, and coordination with primary care/school-based health resources where available. While our dataset cannot identify which barriers dominate, the pattern of repeat adolescent births underscores the importance of continuity of care beyond a single counseling encounter.

Socioeconomic factors such as low educational attainment, rural residence, and limited family support remain central determinants of adolescent pregnancy. While support services (e.g., psychological, social work) were available in our setting, uptake and long-term follow-up remain areas for improvement.

Policy analyses in Romania underline several actionable gaps relevant to adolescent pregnancy prevention: inconsistent standards and clinical pathways, weak cross-sector coordination, limited adolescent-friendly confidential services, uneven school-based education program delivery, and insufficient indicator-based monitoring. Our findings support the need for structured, adolescent-centered care pathways—particularly postpartum counseling coupled with facilitated access to contraception and follow-up—implemented in alignment with national policy recommendations [UNICEF policy framework, 2022].

Several limitations should be acknowledged. First, this is a single-center, retrospective study conducted in a tertiary maternity hospital; therefore, selection and referral patterns may over-represent complicated pregnancies and may not reflect the distribution of adolescent pregnancies at the population level. As a result, our findings should be interpreted as descriptive for this clinical setting and its catchment area rather than as national prevalence estimates. Second, the retrospective design restricts the scope of available variables, particularly for socioeconomic determinants that are not routinely recorded, which limits causal inference regarding upstream drivers of adolescent pregnancy.

## 5. Conclusions

Adolescent pregnancy remains a persistent and multifactorial public health issue in Romania, reflecting the intersection of educational disadvantage, limited healthcare access, and deep-rooted social inequities. Our retrospective analysis of 637 adolescent mothers revealed that low maternal education, rural residence, and inadequate prenatal monitoring were significantly associated with poorer neonatal outcomes, particularly lower birth weights and increased perinatal vulnerability. Although the mean age at first birth showed a modest but significant increase in 2025 compared to previous years, this improvement is insufficient to offset the overall upward trend in adolescent deliveries observed in our institution.

These findings highlight that adolescent pregnancy is not solely a medical phenomenon but a social determinant of health, perpetuating cycles of poverty, limited educational attainment, and early motherhood. Full prenatal monitoring was linked to better neonatal outcomes, underscoring the necessity of accessible, continuous, and adolescent-friendly healthcare services. However, more than two-thirds of the mothers in our study received no or only partial prenatal care, reflecting significant barriers in the healthcare system.

Addressing adolescent pregnancy requires comprehensive, multisectoral strategies: implementing age-appropriate sexual education in schools, improving contraceptive accessibility, ensuring confidentiality in reproductive health services, and strengthening social support systems for young mothers. Without these measures, adolescent pregnancy will continue to contribute to intergenerational cycles of socioeconomic vulnerability and adverse maternal and neonatal outcomes in Romania.

These findings emphasize the need for adolescent-centered preventive strategies and postpartum follow-up pathways that are feasible in routine care. Given the single-center, retrospective design, the results should be viewed as clinically informative for this setting and as a basis for future multi-center or population-based research that can incorporate broader socioeconomic determinants.

## Figures and Tables

**Table 1:** Descriptive characteristics of the study sample (N = 637).

Variable	Mean (SD) or *n* (%)	Minimum	Maximum
Maternal age (years)	17.26 (1.50)	12	19
Gestational age at admission (weeks)	38.03 (2.21)	24	42
Newborn birth weight (grams)	3077.03 (560.80)	670	4550
APGAR score 1 min under7/≥ 7	8.15 (1.61)	2	10
Premature birth (<37 weeks)	91 (14.29%)	24	36
Intrauterine growth restriction (yes)	61 (9.58%)		
Amnionitis (yes)	35 (5.49%)		
Cesarean section (yes)	220 (34.54%)		
Environment (urban)	111 (17.43%)		
Episiotomy (yes)	281 (67.39%)		
Oxytocin administration (yes)	292 (45.84%)		
Anemia before birth (yes)	171 (26.84%)		
Hypertension during pregnancy (yes)	21 (3.30%)		
Leukocytosis (yes)	73 (11.46%)		
Antibiotic administration (yes)	106 (16.64%)		

**Table 2:** Gravidity–parity distribution patterns in a clinical cohort of 204 women who completed at most primary school.

At Most Primary School		Parity				Total
		1	2	3	4	
Gravidity	1	144				144
	2	6	39			45
	3	1	2	9		12
	4		1	1	1	3
Total		151	42	10	1	204

## Data Availability

The raw data supporting the conclusions of this article will be made available by the authors on request.
